# Exploring the Influence of EGCG on the β-Sheet-Rich Oligomers of Human Islet Amyloid Polypeptide (hIAPP_1–37_) and Identifying Its Possible Binding Sites from Molecular Dynamics Simulation

**DOI:** 10.1371/journal.pone.0094796

**Published:** 2014-04-16

**Authors:** Qianqian Wang, Jingjing Guo, Pingzu Jiao, Huanxiang Liu, Xiaojun Yao

**Affiliations:** 1 School of Pharmacy, Lanzhou University, Lanzhou, China; 2 State Key Laboratory of Applied Organic Chemistry and Department of Chemistry, Lanzhou University, Lanzhou, China; 3 State Key Laboratory of Quality Research in Chinese Medicine, Macau Institute for Applied Research in Medicine and Health, Macau University of Science and Technology, Taipa, Macau, China; University of Akron, United States of America

## Abstract

EGCG possesses the ability of disaggregating the existing amyloid fibrils which were associated with many age-related degenerative diseases. However, the molecular mechanism of EGCG to disaggregate these fibrils is poorly known. In this work, to study the influence of EGCG on the full-length human islet amyloid polypeptide 1–37 (hIAPP_1–37_) oligomers, molecular dynamics simulations of hIAPP_1–37_ pentamer and decamer with EGCG were performed, respectively. The obtained results indicate that EGCG indeed destabilized the hIAPP_1–37_ oligomers. The nematic order parameter and secondary structure calculations coupled with the free-energy landscape indicate that EGCG broke the initial ordered pattern of two polymers, greatly reduced their β-sheet content and enlarged their conformational space. On this basis, three possible target sites were identified with the binding capacity order of S1>S2>S3. After a deeper analysis of each site, we found that S1 was the most possible site on which residues B-Ile26/Ala25, A-Phe23, B/C-Leu27 and E-Tyr37 played an important role for their binding. The proposal of this molecular mechanism can not only provide a prospective interaction figure between EGCG and β-sheet-rich fibrils of hIAPP_1–37_, but also is useful for further discovering other potential inhibitors.

## Introduction

Amyloidogenesis plays a critical role in a broad range of different human neurodegenerative diseases such as Huntington, Alzheimer's, Parkinson and type-2 diabetes, all of which are characterized by the pathological deposition of amyloid plaques [1]–[3]. Studies have revealed that insoluble amyloid fibrils formed by diverse protein sequences shared a highly ordered cross-β-sheet pattern, indicating that they had common structural features and similar external morphologies [4], [5]. Although many kinds of intermediates with various morphologies and sizes emerge during the formation of amyloid fibrils [6], it's unclear which of the species (monomers, oligomers, globulomers, protofibrils or fibrils) induce the largest toxicity to neurosomes. However, the converging evidence has suggested that small amyloidogenic oligomers were likely to be most harmful to nerves rather than mature fibrils [7], [8]. It's well known that amyloid formation generally occurs via a nucleated growth mechanism. That's to say, once a nucleus is formed, fibril growth proceeds rapidly by further association of either monomers or oligomers [9]–[12]. Consequently, it's extremely urgent to discover the drugs of inhibiting the formation of amyloid aggregates (especially oligomers).

Recently, various types of inhibitors including β-sheet breaker peptides [13], [14], proteins [15]–[17] and small organic molecules [18]–[22] have been endeavored for their ability to reduce amyloid cytotoxicity. Among them, an important class composed of polyphenols gained much more particular attention than others. As the most abundant biologically active compound in green tea, (-)-epigallocatechin-3-gallate (EGCG) can inhibit the fibrillation of a range of amyloidogenic peptides [23]–[25] including islet amyloid polypeptide, Aβ and α-synuclein by binding to native-unfolded polypeptides and preventing their conversion into toxic, on-pathway aggregation intermediates [24]–[26]. EGCG recently has even been shown to disaggregate the existing fibrils under bulk conditions [23], [27]–[29], which could relieve pathological symptoms and bring hope to patients with the neurodegenerative disease. As a matter of fact, not only fast transition nature but heterogeneous conformations of amyloid aggregates make it extremely challenging to capture and characterize the structural property of peptide or inhibitor-peptide via conventional experimental methods. Therefore, the lacking of high-resolution atomic structures has already been the bottleneck to study the amyloid assembly.

However, computational techniques fill the gap between the research need and the lacking of real structures [30], [31]. To date, a large number of structural details for different amyloid species (monomer, oligomers and fibrils) [32]–[38] and their complex with EGCG [39] or other polyphenols [21], [40], [41] have been achieved. For example, Sun et al. [39] elucidated the molecular mechanism of inhibition effect of EGCG on the conformational transition of Aβ 42 monomer using all-atom molecular dynamics simulation. Recently, Mu et al. [42] have further investigated the molecular mechanism of the inhibition of EGCG on the Aβ 42 dimer using extensive replica-exchange molecular dynamics simulation and the results showed that Aβ dimer with EGCG adopted new conformations, affecting its propensity to adopt fibril-prone states. Although EGCG is believed to be the most potential polyphenol drug, most of current computational studies focus on the inhibition of EGCG on different low aggregate formations of various amyloid peptides. To the best of our knowledge, there is very few aiming at the disaggregating capacity of EGCG on the formed aggregates.

**Figure 1:**
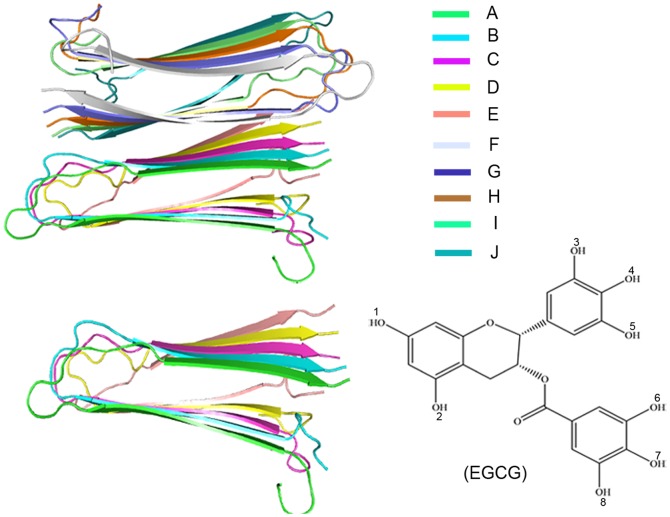
Molecular models of hIAPP_1–37_ pentamer and decamer (Left), and molecular structure of EGCG (Bottom right). The different colors of color-bar account for different chains.

EGCG (Figure 1), as the first small molecule to disaggregate human islet amyloid polypeptide (hIAPP) fibrils [23], has been shown to be very effective to alleviate type-2 diabetes induced from the misfolding and aggregation of hIAPP. IAPP is a member of the calcitonin-like family of peptides existing in all mammalian species [43]–[45]. Once the 37-residue polypeptide misfolds and aggregates, the assemblies will be toxic to pancreatic β-cell by membrane surface which has been proved the cause of type-2 diabetes disease [46]–[51]. Although hIAPP is extremely amyloidogenic, fewer inhibitors for its amyloid formation have been reported than that for Aβ's or αS's.

In this paper, we will concentrate on exploring the disaggregation mechanism of EGCG on the formed aggregates of human islet amyloid polypeptide (hIAPP). Two different sizes of hIAPP_1–37_ oligomers, pentamer (5-mer) and decamer (10-mer), are used (Figure 1). Our aims are to investigate whether EGCG can disaggregate hIAPP_1–37_ oligomers, search for the possible binding sites, and further reveal the important residues by all-atom molecular dynamics simulations. The proposal of molecular mechanism of EGCG's disaggregating the formed hIAPP_1–37_ oligomers will give valuable information for the design and discovery of amyloid inhibitors.

## Materials and Methods

### Molecular Model Built

Initial atomic coordinates of hIAPP_1–37_ 10-mer were kindly provided by the Tycko lab based on the solid-state NMR methods [52]. The hIAPP_1–37_ 5-mer was extracted from the former. Each monomer has a β-strand−loop−β-strand (U-bend) fold consisting of two antiparallel β-strands connected by one turn [β-strand (Lys1-Val17) −turn (His18-Leu27) −β-strand (Ser28-Tyr37)]. There is an intramolecular disulfide bond between Cys2 and Cys7 to stabilize the N-terminus of hIAPP_1–37_. EGCG was drawn manually using Discovery Studio 2.5.5 [53]. The CHARMM topology of EGCG was generated by the ParamChem Sever (https://www.paramchem.org/index.php), and the partial atomic charges and charge groups of EGCG were assigned as a previous study did [39]. Names and types of atoms, charges and masses of EGCG were given in Table S1 in the Supporting Information. In order to unclose the mechanism clearly, both hIAPP_1–37_ 5-mer and 10-mer were simulated in 0.18 mol L^−1^ EGCG (EGCG/hIAPP_1–37_ 5-mer = 7; EGCG/hIAPP_1–37_ 10-mer = 10). The concentration ratios of EGCG to hIAPP_1–37_ were within the general experimental range *in vitro* (from 1∶1 to 22∶1) [54], [55]. Seven and ten EGCG molecules were then placed randomly around hIAPP_1–37_ 5-mer and hIAPP_1–37_ 10-mer, respectively.

### Simulation Details

All the molecular dynamics (MD) simulations were performed using NAMD 2.9b3 program [56] together with CHARMM22 force field [57] and explicit solvent model under periodic boundary conditions. Initially, the complexes of EGCG with hIAPP_1–37_ 5-mer and 10-mer were placed in the 80 Å *80 Å *100 Å box and 90 Å*100 Å*100 Å box with TIP3P water model [58], respectively. To keep the system neutral, 10 and 20 chloride ions were added into the systems of 5-mer and 10-mer, respectively. Initial configurations were minimized in three steps, first keeping the protein and EGCG constrained, and then only keeping the protein backbone constrained and finally keeping all of molecules free. These were followed by 500 ps equilibration. For our each system, a total of 200 ns MD simulation was performed without any restraints in the isothermal isobaric (NPT) ensemble with 310 K and 1 bar pressure, and the obtained atomic coordinates were saved every 1 ps for analysis. As control systems, both hIAPP_1–37_ 5-mer and hIAPP_1–37_ 10-mer without EGCG were also simulated with the similar procedure above.

During the molecular dynamics simulation, the Langevin dynamics was used to control the temperature and the Langevin damping coefficient was set to 5 ps. The Langevin piston Nose-Hoover method was applied to keep the pressure constant. Long-range electrostatic interactions were computed using particle mesh Ewald method [59], and van der Waals interactions were smoothly switched off in the interval from 10 to 12 Å. All the bonds involving hydrogen atom were considered to be rigid using SHAKE algorithm [60], allowing an integration time step of 2 fs.

### Trajectory Analysis

The nematic liquid crystalline order parameter (*P2*), discriminating between ordered and disordered conformations, has been used to characterize the fibril-like state of peptides and show the overall orientation of peptides with respect to a director [61], [62]. *P2* value is calculated by
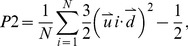
(1)where 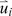 is a unit vector linking N and C terminus for the *i*th peptide, 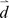
(the director) is a unit vector defining the preferred direction of alignment, and *N* is the number of molecules. Here, the cut-off value of *P2* is set to 0.5 as ever used by Li's study [14], and the system is considered as the ordered state if *P2* value exceeds 0.5.Recently, free energy landscape (FEL) has been widely used in the study of amyloid system [14], [61], [63]. The free-energy surface along the N-dimensional reaction coordinate 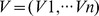 is given by
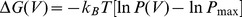
(2)Here, *P(V)* is the probability distribution from MD data and Pmax denotes the maximum of the distribution which is subtracted to ensure that Δ*G* = 0 for the lowest-free-energy minimum [64]. 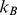 is the Boltzmann constant. Generally, principal components analysis (PCA) can be used to obtain the first two eigenvectors corresponding to two largest eigenvalues, and these two eigenvectors can further be used as the reaction coordinates to construct the free-energy landscape. Here, given that the β-sheet content and the gyration radius (Rg) were closely related to fibril formation [14], we employed them as reaction coordinates of the two-dimensional FEL.

It's well known that hydrogen bond (H-bond) interaction especially backbone hydrogen bond plays a key role in the amyloid assembly and is used frequently to monitor the fibril formation. A hydrogen bond is considered to be formed if the distance between donor D and acceptor A is less than 3.5 Å and the D-H-A angle is larger than 150^o^. Additionally, a sidechain or backbone contact is considered to be formed if an atom of one residue is within 3.5 Å around one random atom of EGCG. STRIDE algorithm [65] then is applied to determine the secondary structure. The definition of secondary structure in STRIDE is based not only on dihedral angles φ and φ but also on hydrogen bond. Hence, this algorithm is more accurate for our systems than that based purely on geometrical constrains.

## Results and Discussion

### Convergence Assessment

**Figure 2:**
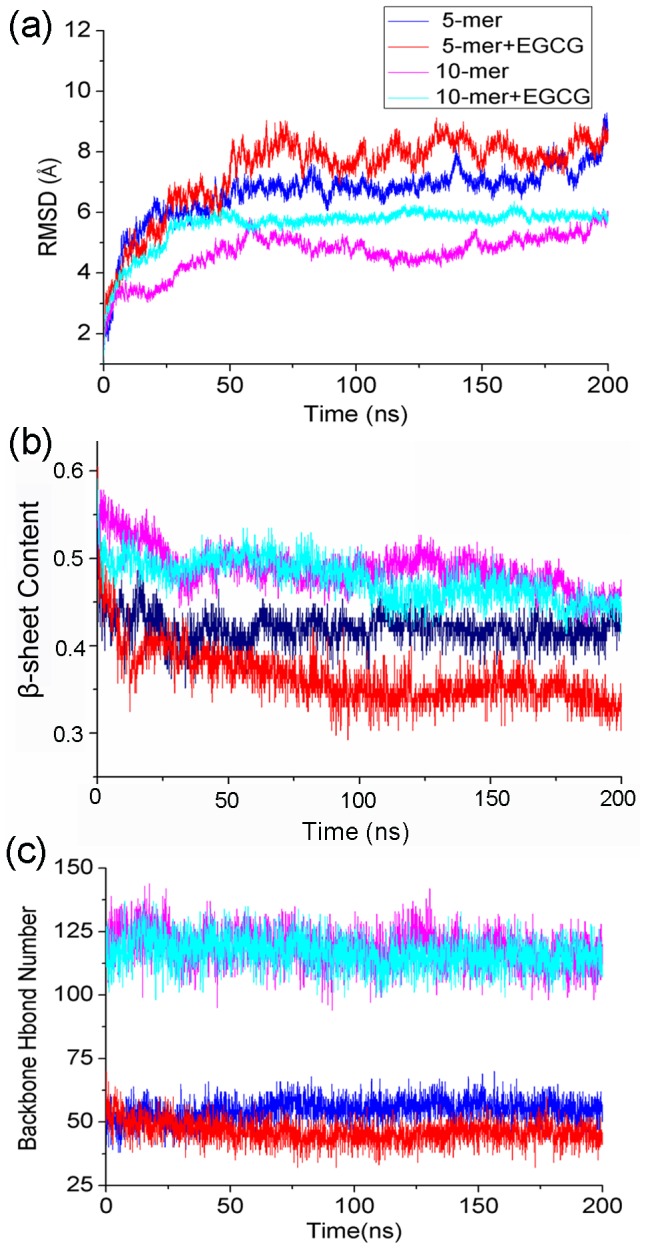
Time dependences of structural characteristics for hIAPP_1–37_ oligomers in the absence or presence of EGCG: a) RMSD; b) β-sheet content; c) backbone hydrogen bond number.

The convergence of MD simulations was herein monitored by three variables. Firstly, we calculated the root-mean-square deviation (RMSD) of Cα atoms of hIAPP_1–37_ oligomers from the initial structure to quantify the conformational changes of this protein during the 200 ns trajectory (Figure 2a). From Figure 2a, it's notable that RMSD values of four systems go up rapidly during the first 50 ns simulation, whereas fluctuate stably in the last 150 ns. Two systems of hIAPP_1–37_ 5-mer (with and without EGCG) have the wider range of fluctuations than that of hIAPP_1–37_ 10-mer, indicating that the former has larger conformational changes than the latter. For the hIAPP_1–37_ 5-mer without EGCG, we note that there is a continuous increase of RMSD values at the last 13 ns. By further analyzing the conformational changes for the last 13 ns and the rest equilibrated trajectory, we found that the obvious structural changes were mainly derived from the flexible N-terminal loop (residues 1–10) of edge chain A. But, the free hIAPP_1–37_ 5-mer keeps the ordered state and β-sheets are intact all the time even at the last 13 ns. This is easy to understand since the loop has large flexibility generally and the terminal loop of the edge chain in a single layer of oligomer has no stable factor like the loop of middle chain. Then, β-sheet content of hIAPP_1–37_ oligomers in all runs was monitored along the simulation time (Figure 2b). Here, we defined the β-sheet content as the ratio of the number of residues in β secondary structure to the total number of residues (37 in this study). As can be seen in Figure 2b, during the first 100 ns simulation, β- sheet contents for all the systems have the fluctuations on different levels, but remain stable in the last 100 ns, suggesting that the simulations are up to equilibrium at 100 ns. The averaged β-sheet contents for all the systems are ranked as 10-mer, 10-mer+EGCG, 5-mer and 5-mer+EGCG, which indicates that 10-mer is more stable than 5-mer and that EGCG does perturb the structural stability of initial hIAPP_1–37_ oligomers by reducing their β-sheet content.

Generally, amyloid fibrils are formed by peptides in extended or β-sheet conformation through parallel or antiparallel backbone hydrogen bonding bridges, which further stack tightly through steric effects at a completely dry interface, called a zipper [66]. Therefore, the formation and stability of amyloid fibrils are closely related to peptide-peptide backbone hydrogen bonds. In our systems, β-sheets between monomers in a single layer are parallel, and one residue hence can form parallel backbone H-bonds with another one in the same direction. Here, we calculated the total backbone H-bond number to characterize the overall change of inter-peptide interactions. As viewed in Figure 2c, H-bond number remains stable in a very small range except the obvious fluctuation in the initial 80 ns in each system. It also can be seen that the ranking order of the averaged H-bond numbers for four simulations agrees well with that of β-sheet content (Figure 2b), suggesting that the formation of backbone hydrogen bonds are closely related to the stability of oligomers, and that the changes of backbone H-bonds can reflect the change of order degree of β-sheet oligomers partly.

Taken together, all these results indicate that the conformational ensembles converged from 100 ns. Thus, the subsequent analysis was based on the last 100 ns trajectory.

### Overall Structural Changes of hIAPP_1–37_ Oligomers Induced by EGCG

**Figure 3:**
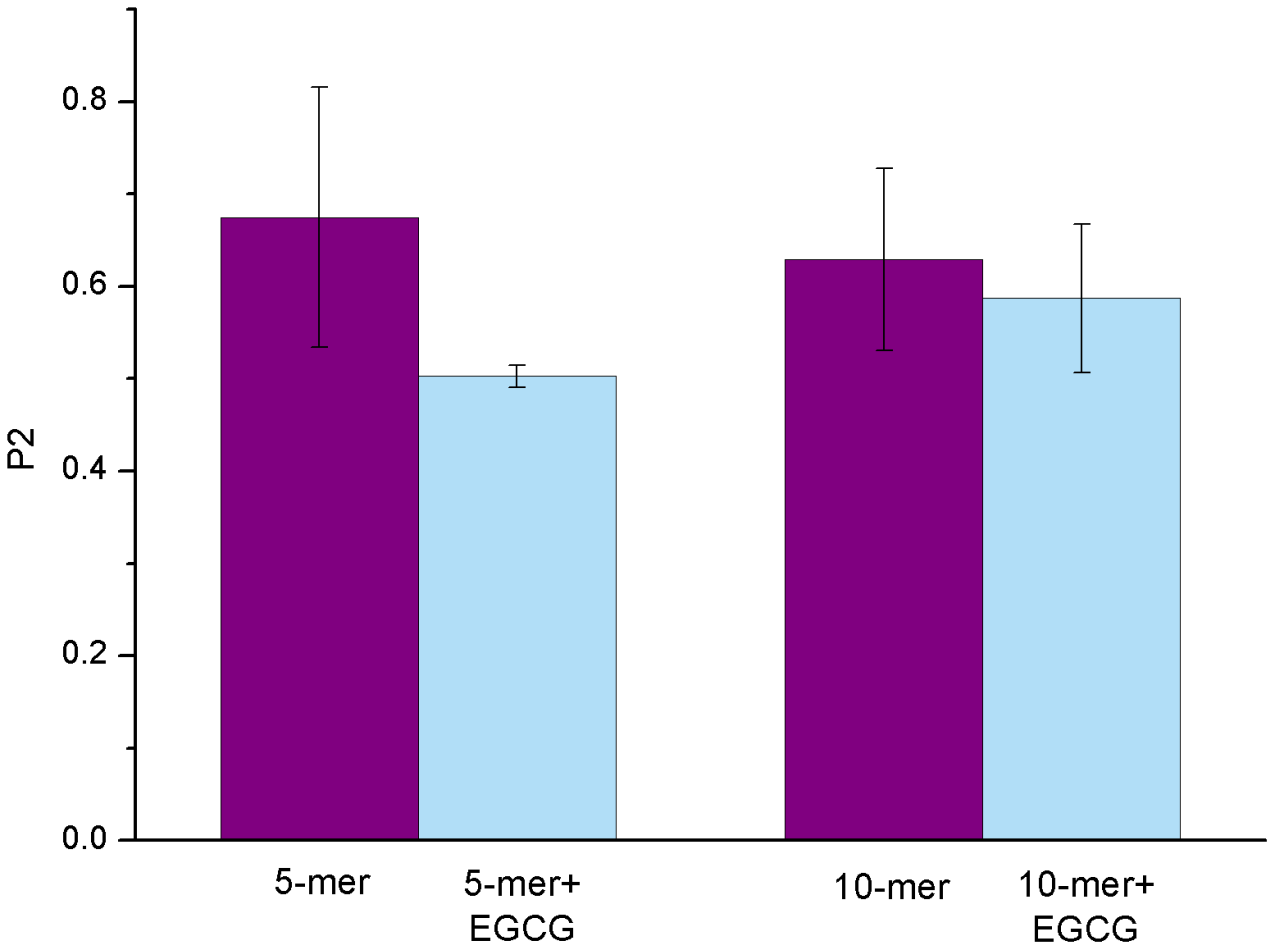
The ordered degree of overall oligomers measured by the averaged *P2* value for each system. The error bar represents the standard deviation.

The nematic order parameter (*P2*) is often used to describe the orientational order of the system and discriminate between ordered and disordered conformations [61], [62]. Here, we applied it to characterize the order degree of hIAPP_1–37_ oligomers. The obtained results were shown in Figure 3. From Figure 3, it can be seen that *P2* value of hIAPP_1–37_ 5-mer without EGCG is larger than 0.6, which means that this polymer maintains the ordered conformation. However, in the presence of EGCG, *P2* value of hIAPP_1–37_ 5-mer decreases around 0.5, meaning that this polymer becomes disordered and unstable by induction of EGCG. As for the hIAPP_1–37_ 10-mer system with EGCG, although *P2* value exceeds 0.5, there's still an obvious reduction relative to that without EGCG, suggesting that EGCG can disturb the stable hIAPP_1–37_ 10-mer structure. In addition, the reduction of *P2* value upon the binding of EGCG in 10-mer system is smaller than that in 5-mer system, indicating that hIAPP_1–37_ 10-mer has higher stability than 5-mer.

**Figure 4:**
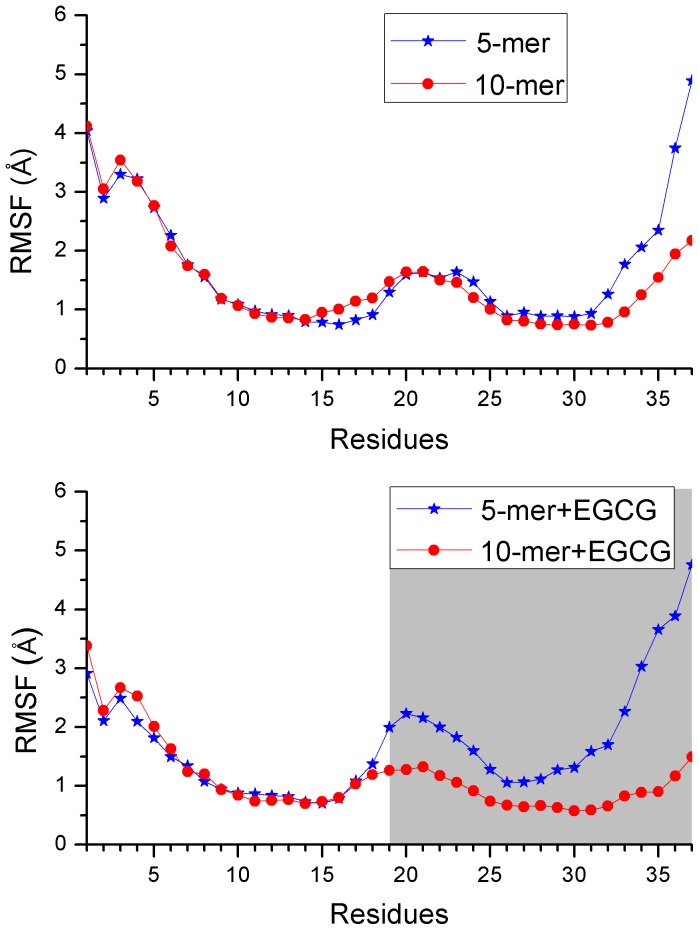
The averaged Cα RMSFs for each chain of hIAPP_1–37_ oligomer as a function of residues.

To explore the details of structural changes, the root-mean-square fluctuation (RMSF) and secondary structural analysis were performed. Firstly, the root-mean-square fluctuations of Cα atoms (Cα RMSFs) of hIAPP_1–37_ for the last 100 ns trajectory were calculated and further averaged for each chain of oligomers. The averaged Cα RMSFs of each residue for different systems were shown in Figure 4. As can be seen, in the absence of EGCG, Cα RMSFs of hIAPP_1–37_ 5-mer fluctuate towards the similar tendency with that of hIAPP_1–37_ 10-mer, indicating that backbone flexibilities of two oligomers are similar under the stable condition. However, once EGCG is added, there's something different. Residues 1–18 still have almost identical fluctuations for two oligomers, whereas the fluctuations of residues 19–37 have obvious difference. The notable distinction of RMSFs at residues 19–37 suggests that EGCG may interact with hIAPP_1–37_ around this domain, and that hIAPP_1–37_ 5-mer is less stable than hIAPP_1–37_ 10-mer.

**Figure 5:**
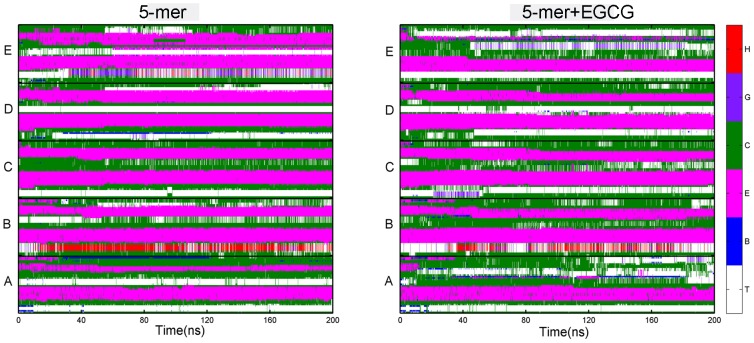
Time evolutions of the secondary structure of hIAPP_1–37_ 5-mer calculated by STRIDE algorithm. Here, we label a turn by “T”, an isolated bridge by “B”, an extended conformation by “E”, a coil by “C”, a 3_10_-helix by “G” and an α-helix by “H”.

As we know, the changes of ordered state of an oligomer correlate closely with the changes of secondary structure. We then calculated the contents of different secondary structures of hIAPP_1–37_ pentamer and decamer. As for hIAPP_1–37_ 5-mer, the presence of EGCG resulted in the great reduction of β-sheet content with the averaged value from 41.5% to 34.0% (Table 1). Meanwhile, the contents of turn and coil increased from 20.0% to 24.8% and 35.7% to 39.0%, respectively, suggesting that β-sheet structure transformed into the disordered structure. As for hIAPP_1–37_ decamer, there are similar change trends of secondary structure to hIAPP_1–37_ pentamer but on less degree (Table 1). By monitoring the evolution of the secondary structure of each residue over time (shown in Figure 5 and Figure S1), it can be seen that the missing of β-sheet mainly occurred at C-terminal of each chain after EGCG is added, which agrees well with the obvious difference of RMSFs at residues 19–37 above. The evolution of secondary structures of both oligomers also suggests that EGCG may act as a β-sheet breaker by the content of β-sheet structure.

**Table 1:** The averaged secondary structure contents of hIAPP_1–37_ in different simulation systems.

Secondary structures	Coil(%)	Turn(%)	β-sheet(%)	Helix(%)
5-mer	35.7(0.02)	20.0(0.03)	41.5(0.01)	1.6(0.01)
5-mer+EGCG	39.0(0.03)	24.8(0.04)	34.0(0.01)	0.1(0.01)
10-mer	13.6(0.01)	12.2(0.01)	23.5(0.01)	0.1(0.003)
10-mer+EGCG	16.3(0.01)	9.2(0.01)	22.4(0.01)	0.7(0.005)

Data is calculated based on the last 100 ns trajectory. The numbers in parentheses are standard deviations.

**Figure 6:**
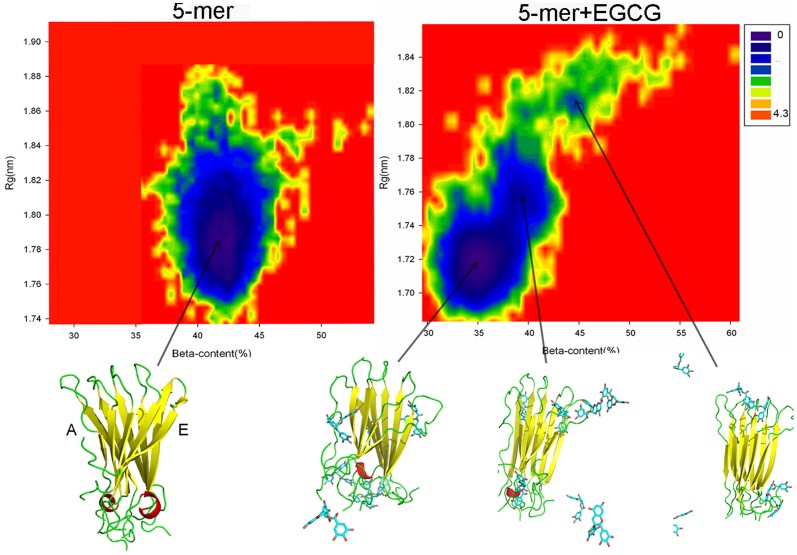
Free energy landscapes (in kcal mol^−1^) and lowest-free-energy structures at basins for hIAPP_1–37_ 5-mer in the presence and absence of EGCG.

Additionally, free energy landscape can not only show the changes of conformational space of a protein during the MD simulation but also be conveniently used to search for the preferred conformation with the low free energy. For our systems, β-sheet content and the gyration radius (R_g_) were used as reaction coordinates to construct the two-dimensional free-energy landscape (FEL) [14]. From Figure 6, the FEL of hIAPP_1–37_ 5-mer without EGCG has only one big basin (center coordinate: (42.5%, 1.78)) corresponding to the lowest free energy, which indicates that 5-mer maintains the overall structural stability along the entire simulation. However, in the presence of EGCG, two additional local basins (center coordinate: (39.0%, 1.75), (44.5%, 1.81)) emerge except the global basin (center coordinate: (35.0%, 1.72)). The relatively dispersive conformational space and new basins for hIAPP_1–37_ 5-mer with EGCG mean that the conformational space of this oligomer indeed altered notably and became more diverse. Moreover, by comparing the center coordinates of global basins in hIAPP_1–37_ 5-mer without and with EGCG, we found that the reduction of beta-sheet content from 42.5% to 35.0% was consistent well with the result of secondary structural analysis, indicating that the addition of EGCG did make hIAPP_1–37_ 5-mer more disordered and unstable. In order to give the information of conformational changes visually, the corresponding structures with the minimum free energy were further extracted. In Figure 6, the presence of EGCG resulted in that the whole β-sheet of chain A of hIAPP_1–37_ 5-mer disappeared, and chain E only had a few residues in β-sheet secondary structure. This behavior further implies the distinction of two lateral environments of this single layer, which has recently been point out [67]. The FEL figure of 10-mer system (Figure S2) is similar to that of 5-mer though β-sheet of chain A is not broken completely.

From the above analysis, small molecular inhibitor, EGCG, can reduce the structural stability of hIAPP_1–37_ oligomers and convert their ordered structure partly into the disordered, which is reflected from the obvious decreasing of beta-sheet content. Additionally, the above analysis also shows that hIAPP_1–37_ 5-mer has much more obvious conformational changes than hIAPP_1–37_ 10-mer, which may be caused by the higher inner stability of hIAPP_1–37_ 10-mer than 5-mer. This result agrees with that the stability of hIAPP oligomer increases along with the number of peptides by Zheng et al [68]. Thus, the following disaggregation mechanism analysis of EGCG mainly focuses on the hIAPP_1–37_ 5-mer.

### The Disaggregation Mechanism of EGCG on hIAPP_1–37_ Oligomer

**Figure 7:**
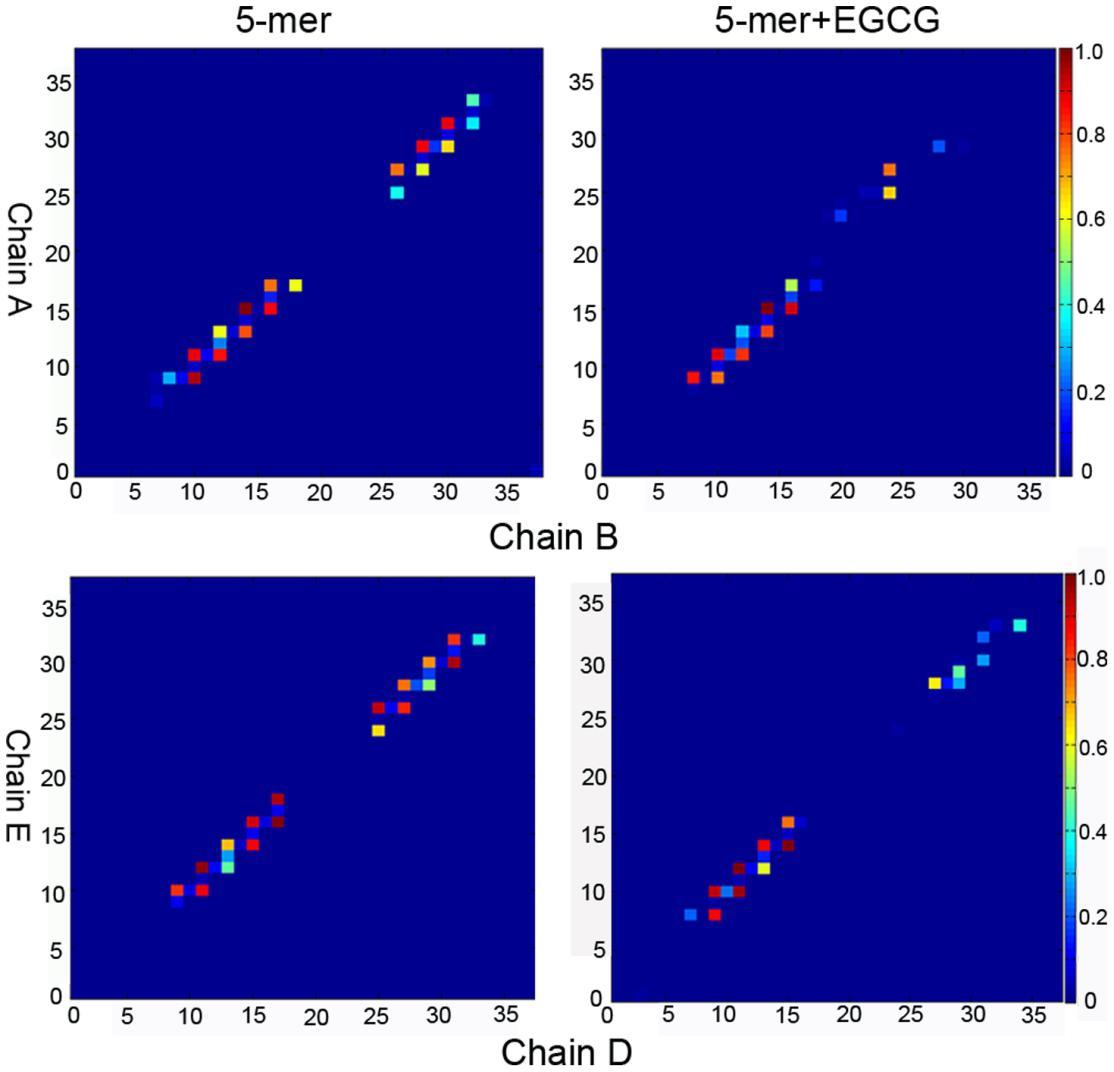
H-bond maps of chain A and B, and chain D and E of hIAPP_1–37_ 5-mer in the presence and absence of EGCG.

How does EGCG break the β-sheet structure of oligomer? From the analysis of Figure 2b, we know the formation of backbone hydrogen bonds are closely related to the stability of oligomers, and that the changes of backbone H-bonds can reflect the change of order degree of β-sheet oligomers partly. So here, to understand the disaggregation mechanism of EGCG on hIAPP_1–37_ oligomer, we further analyzed the changes of native backbone H-bonds of hIAPP_1–37_ oligomer upon binding to EGCG. Since the disaggregation of oligomers begins from edge chains, we only focus on the edge chains (chain A/B and chain D/E) of hIAPP_1–37_ 5-mer. Based on the statistic of H-bond numbers, the backbone H-bond maps were plotted in Figure 7 by setting the largest H-bond number as 1 for reference in each group. Figure 7 shows that the backbone H-bond number between chain A and B in hIAPP_1–37_ 5-mer in the presence of EGCG is notably lower than that without EGCG, indicating that native inter-peptide H-bond pattern of hIAPP_1–37_ 5-mer is broken by EGCG. The relatively large H-bond reduction occurring at C-terminal β-strand suggests that this region is broken more seriously than the N-terminal β-strand, and may be the main interaction region of EGCG. The similar phenomenon happens at the other edge, chain D and E.

To gain the interaction details between EGCG and hIAPP_1–37_ oligomer, cluster analysis was further performed to extract the representative conformations using the last 100 ns trajectory of hIAPP_1–37_ 5-mer with EGCG. Snapshots were collected at 2 ps interval. The conformational clustering based on RMSD with SOM algorithm was carried out using the PTRAJ module of AMBER 10.0 [69]. Prior to clustering, each conformation was superposed onto the initial structure to remove rigid-body motion including the rotation and translation. In order to make the conformational classification more accurate, different sizes of conformational clusters (three, four, five) were then carried out. The consistent results were obtained that the first two classes dominate in the obtained clusters. For example, as for the three conformational clusters, the occupancies of three classes are 44.3%, 42.3% and 13.4%, respectively, indicating that the first two classes can account for most of conformational space. By aligning the representative structures of the first two classes (Figure S3), we observe that the overall hIAPP_1–37_ 5-mer fluctuates slightly, but the positions and conformational changes of EGCG molecules are fairly notable and intense except EGCG molecules on the three sites S1, S2 and S3. Among most time of the last 100 ns, small molecules on these three positions always stay at the corresponding position with a little change. To further prove the reliability of our identified three sites, one parallel 200 ns trajectory for the complex of hIAPP_1–37_ 5-mer and EGCG was additionally simulated. After similar analysis, we found the identified three sites were same in two parallel trajectories (Figure S4). Therefore, these three sites on hIAPP_1–37_ 5-mer may be the possible binding sites of EGCG.

**Figure 8:**
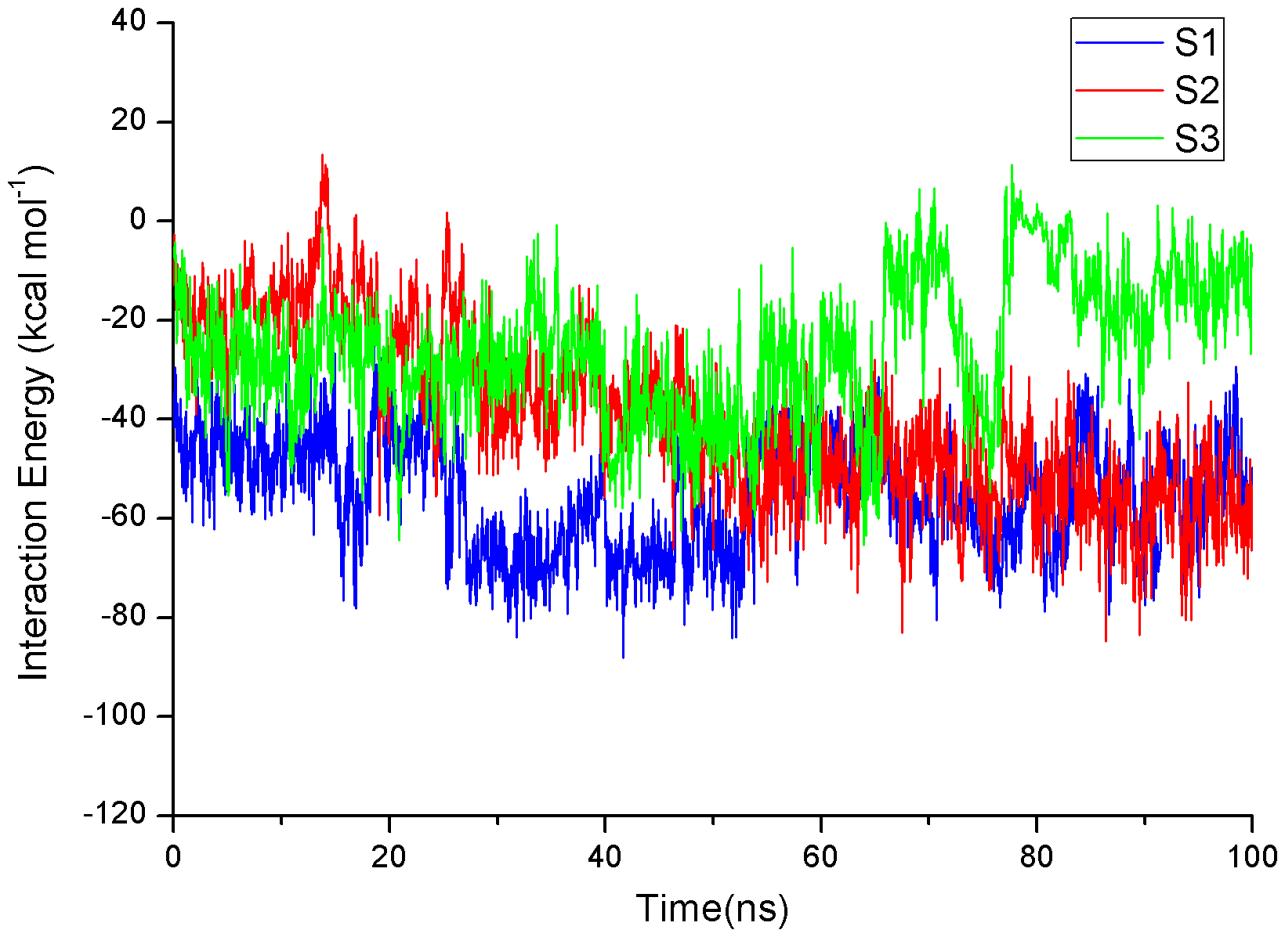
Time evolutions of interaction energy of EGCG with hIAPP_1–37_ 5-mer on three sites based on the last 100 ns trajectory.

To assess roughly their binding affinity at each site, the interaction energy including van der Waals and electrostatic interactions based on the last 100 ns trajectories was calculated by NAMD Energy plugin in VMD 1.9.1 [70]. As shown in Figure 8, the fluctuation of interaction energy at S1 site keeps very small and the averaged interaction energy is lowest (−55.19 kcal mol^−1^) relative to that at S2 site (−39.68 kcal mol^−1^) and S3 site (−25.79 kcal mol^−1^).

**Figure 9:**
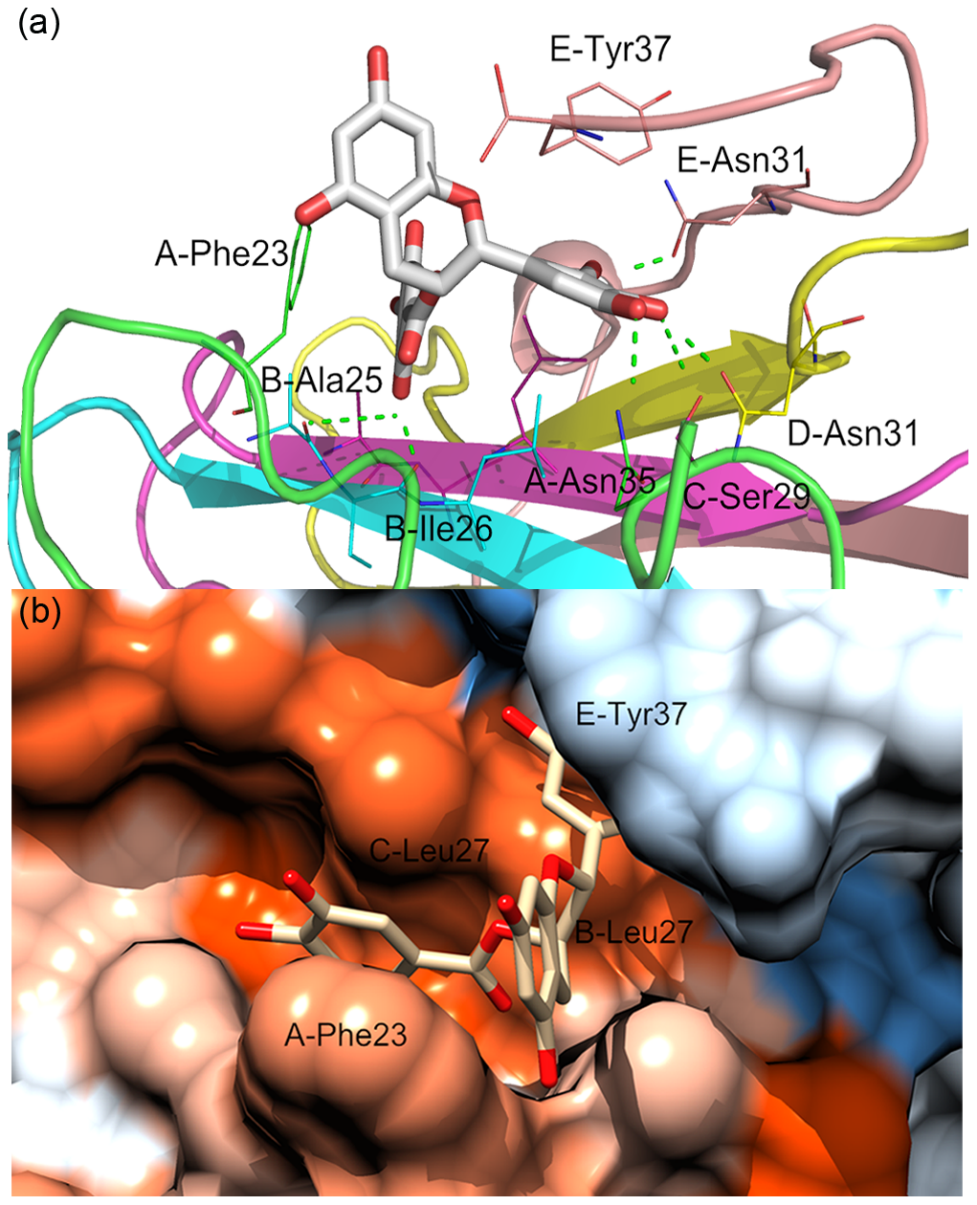
Detailed interaction of EGCG and hIAPP_1–37_ 5-mer on S1 site: a) the static interaction mode of EGCG and S1 site; b) hydrophobic surface of S1 site. The color gradient from blue to orange is indicative of an increase in hydrophobicity.

**Figure 10:**
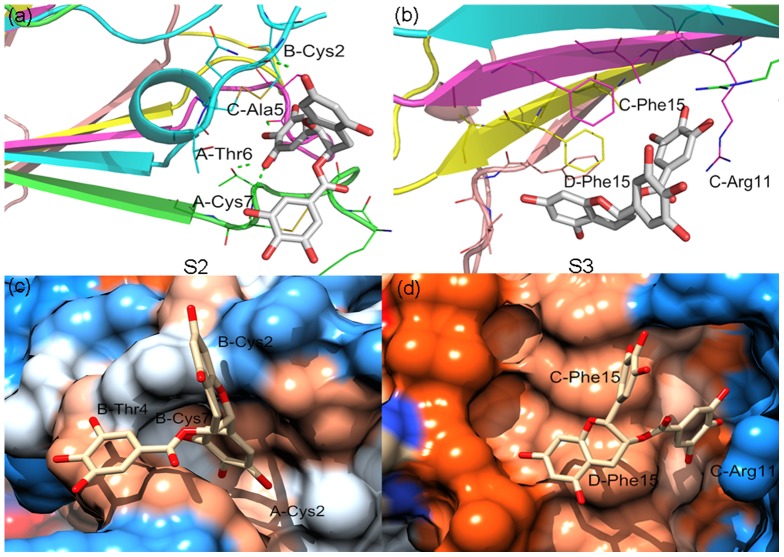
Detailed interactions of EGCG and hIAPP_1–37_ 5-mer on S2 and S3 site: a) the static interaction mode of EGCG and S2 site; b) the static interaction mode of EGCG and S3 site; c) hydrophobic surface of S2 site; d) hydrophobic surface of S3 site. The color gradient from blue to orange is indicative of an increase in hydrophobicity.

Fully understanding the interaction features of EGCG on three sites is helpful for us to identify the real binding site and understand the disaggregation mechanism of EGCG. The static detailed interactions between EGCG and three possible binding sites were shown in Figure 9 and 10. This representative structure was obtained by extracting the first class structures of the above cluster analysis. Here, for clarity, residues were marked in a simplified way. For instance, A-Phe23 is referred to residue Phe23 of chain A.

As for S1 site, from Figure 9a, it's evident that EGCG forms six hydrogen bonds with different residues of hIAPP_1–37_ 5-mer. They exist between the hydroxy group at the 8 position of EGCG and the main-chain carbonyl groups of B-Ile26/B-Ala25, the hydroxy group at the 4 position and the side-chain carbonyl group of D-Asn31/the hydroxy group of C-Ser29, the hydroxy group at the 3 position and the side-chain carbonyl group of E-Asn31, and the hydroxy group at the 5 position and the side-chain amino group of A-Asn35, respectively. We also calculated the occupied percentage of each hydrogen bond to describe the importance of hydrogen bond. The obtained results indicate that the first four H-bonds are relatively important with the occupied percentages of 37.6%, 16.1%, 23.5% and 20.8%, respectively. Actually, the formation of these H-bonds between EGCG and hIAPP_1–37_ 5-mer has large influence on the inter-peptide backbone H-bonds of oligomer. From Figure 7, we can see that without EGCG, B-Ile26 on S1 site can form obvious backbone H-bond interactions with residues Ala25 and Leu27 of chain A to stabilize the β-sheet oligomer. However, in the presence of EGCG, the backbone of B-Ile26 formed the strong hydrogen bond with EGCG instead of chain A, which broke the original backbone H-bonds of this domain. Similarly, the formation of hydrogen bonds between EGCG and side chains of D-Asn31 and E-Asn31 also has certain influence on the native H-bond pattern of inter-chains in hIAPP_1–37_ 5-mer (Figure 7). In addition, π-π stacking is another important interaction between EGCG and hIAPP_1–37_ oligomers. In this system, there are mainly two groups of π-π stacking interactions. The one locates between benzene rings of Phe23 of chain A and the gallate ester moiety of EGCG, and the other exists between benzene rings of Tyr37 of chain E and the trihydroxyhpenyl of EGCG. Previous studies also have illustrated that aromatic residues (Phe, Trp and Tyr) played a key role in the formation and inhibition of amyloid fibrils (especially Phe23 for hIAPP) [71]–[74].

In order to visualize the hydrophobic interaction of EGCG and hIAPP_1–37_ 5-mer at S1 site, hydrophobic surface of this binding domain was then drawn. As is evident from Figure 9b, we can see that EGCG is buried into a deep hydrophobic pocket which is composed of residues B/C-Leu27, A-Phe23 and E-Tyr37. The "door" of this pocket is controlled by residues A-Phe23 and E-Tyr37, keeping the small molecule stay at it stably. The formation of this pocket disturbed the initial ordered surface of hIAPP_1–37_ 5-mer.

Figure 10a shows that the number of hydrogen bonds at S2 site is less than that at S1 site, and two H-bonds are important which locate between the hydroxy group at the 4 position of EGCG and the main-chain carbonyl group of C-Ala5 (37.8%), and the hydroxy group at the 5 position and the main-chain amino group of A-Cys7 (33.0%), respectively. In this domain, we haven't found obvious π-π stacking interactions like S1 site above. However, we noticed that EGCG split the parallel β-sheets to construct a pocket for itself. The trihydroxyhpenyl of EGCG stretched into the pocket formed by residues B-Thr4/Cys7/Cys2 and A-Cys2, whereas other two benzene rings kept outside. In addition, it can be observed that the hydrophobicity of this domain is lowest in three sites (Figure 10c). For S3 site, the location of EGCG is opposite to that for S1 site, and the distinction of its binding modes with hIAPP_1–37_ at two sites is quietly notable. Firstly, although EGCG can form hydrogen bonds with hIAPP_1–37_ 5-mer at this site during the MD simulation, occupancies of all the H-bonds keep very low and the largest one is 5.8% (the hydroxy group at the 8 position of EGCG and the side-chain amino group of C-Arg11). Conformational analysis shows that there are two perpendicular π-π stacking interactions (Figure 10b). They locate between benzene rings of Phe15 of chain C and gallate ester moiety of EGCG, and Phe15 of chain D and 2H-1-benzopyran-3-yl, respectively. The surface of S3 site is also hydrophobic or even more hydrophobic than others (Figure 10d), but doesn't form the hydrophobic pocket like S1 site. Furthermore, the binding of EGCG at this site does not disturb the N-terminal β-sheets of hIAPP_1–37_ 5-mer, indicating the less possibility of EGCG binding on this site.

From all the above analysis, we conclude that for our studied small-molecule inhibitor EGCG, S1 is its most possible binding site among the initially identified three sites. As we know, the C-terminal amyloidogenic region of hIAPP_1–37_ (especially residues 20–29) is of considerable importance for the formation and growth of amyloid fibrils. The breaking of this domain for the ordered aggregates will have large influence on the lateral stacking of another approaching β-layer during the amyloid growth. Since the most possible S1 site locates around this domain, we think that EGCG may inhibit the growth of hIAPP fibrils by blocking the lateral association, just like another polyphenol inhibitor resveratrol [75].

**Figure 11:**
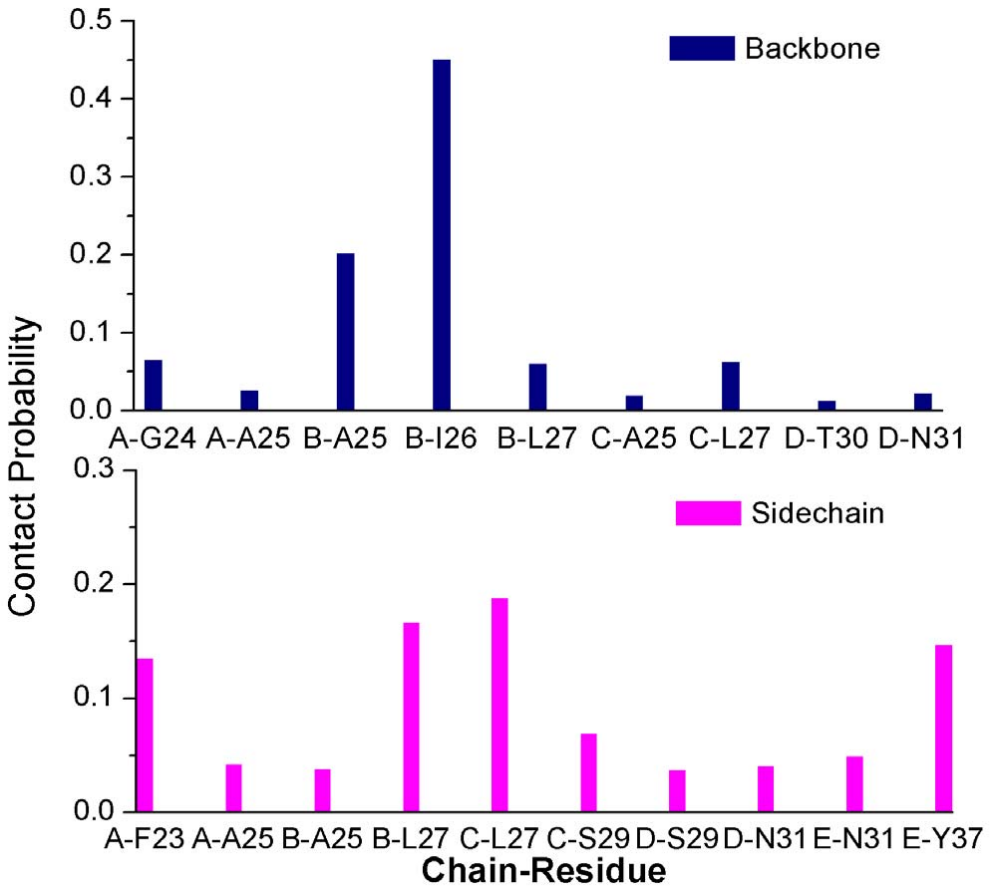
Contact probabilities of EGCG with backbone and sidechain atoms of hIAPP_1–37_ 5-mer on S1 site. For clarity, residues whose contact probabilities were lower than 0.01 were not given.

Next, to identify the key residues on S1 site for the binding of EGCG, atomic contact numbers of EGCG with backbone atoms and sidechain atoms of each residue of hIAPP_1–37_ 5-mer were both calculated based on the last 50 ns trajectory, and the results were shown in Figure 11. If the contact probability of one residue with EGCG is larger than 0.1, this residue is considered to have the important contribution for the binding of EGCG to hIAPP_1–37_. By averaging the contact numbers of EGCG with backbone and sidechain at three binding sites, we found that the sidechain of hIAPP_1–37_ played a more important role than the backbone section (Table 2).

**Table 2:** The averaged contact numbers of EGCG with backbone and sidechain atoms of hIAPP_1–37_ 5-mer on three possible binding sites.

	Backbone	Sidechain
S1	10.58	105.80
S2	12.60	66.00
S3	1.99	42.77

As shown in Figure 11, the main contribution for backbone atoms comes from residues B-Ile26 and B-Ala25. Given the above hydrogen bond analysis that residues B-Ile26 and B-Ala25 have a large probability to form H-bond with EGCG, it can be deduced that backbone atoms of B-Ile26 and B-Ala25 play an important role in the binding of EGCG by forming strong hydrogen bond interactions. Most of the contribution for sidechain atoms comes from residues A-Phe23, B/C-Leu27 and E-Tyr37. In the above binding mode analysis, both A-Phe23 and E-Tyr37 can form aromatic π-π stacking interactions with EGCG. Therefore the sidechain aromatic rings of A-Phe23 and E-Tyr37 do contribute obviously to the binding of EGCG. Leu27 from chain B and chain C together with A-Phe23 and E-Tyr37 construct the hydrophobic pocket of S1 site and also form the strong hydrophobic interaction with EGCG.

## Conclusions

In this study, by performing all-atom molecular dynamics simulations, we investigated the influence of EGCG on the β-sheet-rich oligomers (pentamer and decamer) of human islet amyloid polypeptide (hIAPP). The obtained results show that small molecular inhibitor, EGCG, is indeed able to break the structural stability of hIAPP_1–37_ oligomers and disaggregate them by converting the ordered structure into the disordered. By cluster analysis, we discovered three possible target sites of EGCG on the surface of hIAPP_1–37_ oligomer. After the detailed binding mode analysis, S1 was considered as the most possible one. On S1 site, we found that EGCG formed the strong hydrogen bond, hydrophobic and π-π stacking interactions with hIAPP. In addition, residues B-Ile26, B-Ala25, A-Phe23, B/C-Leu27 and E-Tyr37 play important roles during EGCG binding process. Our work first reveals the disaggregation mechanism of EGCG on the amyloid aggregates, which is helpful for the design and discovery of new drugs with the same disaggregating ability.

## Supporting Information

Figure S1 **Time evolutions of the secondary structure of hIAPP_1–37_ 10-mer calculated by STRIDE algorithm.** Here, we label a turn by “T”, an isolated bridge by “B”, an extended conformation by “E”, a coil by “C”, a 3_10_-helix by “G” and an α-helix by “H”.
(TIF)

Figure S2 **Free energy landscapes (in kcal mol^−1^) and lowest-free-energy structures at basins for hIAPP_1–37_ 10-mer in the presence and absence of EGCG.**
(TIF)

Figure S3 **The aligned representative structures of the first two clusters.** Violet and yellow cartoon corresponds to the representative structure of the first and second cluster, respectively. Three domains circled by dashed line are indicative of S1, S2 and S3 site.
(TIF)

Figure S4 **Time evolutions of RMSD values of hIAPP_1–37_ 5-mer with EGCG for the parallel 200 ns trajectory together with the aligned representative structures of the first two clusters from cluster analysis (last 100 ns).**
(TIF)

Table S1 **Names and types of atoms, charges and masses of EGCG used in the simulations.**
(DOC)
