# Supplementary figures and images for: Exploring the Influence of EGCG on the β-Sheet-Rich Oligomers of Human Islet Amyloid Polypeptide (hIAPP1–37) and Identifying Its Possible Binding Sites from Molecular Dynamics Simulation
Source: PLoS One. 2014 Apr 16;9(4):e94796. doi: 10.1371/journal.pone.0094796 (PMC3989243; doi:10.1371/journal.pone.0094796)

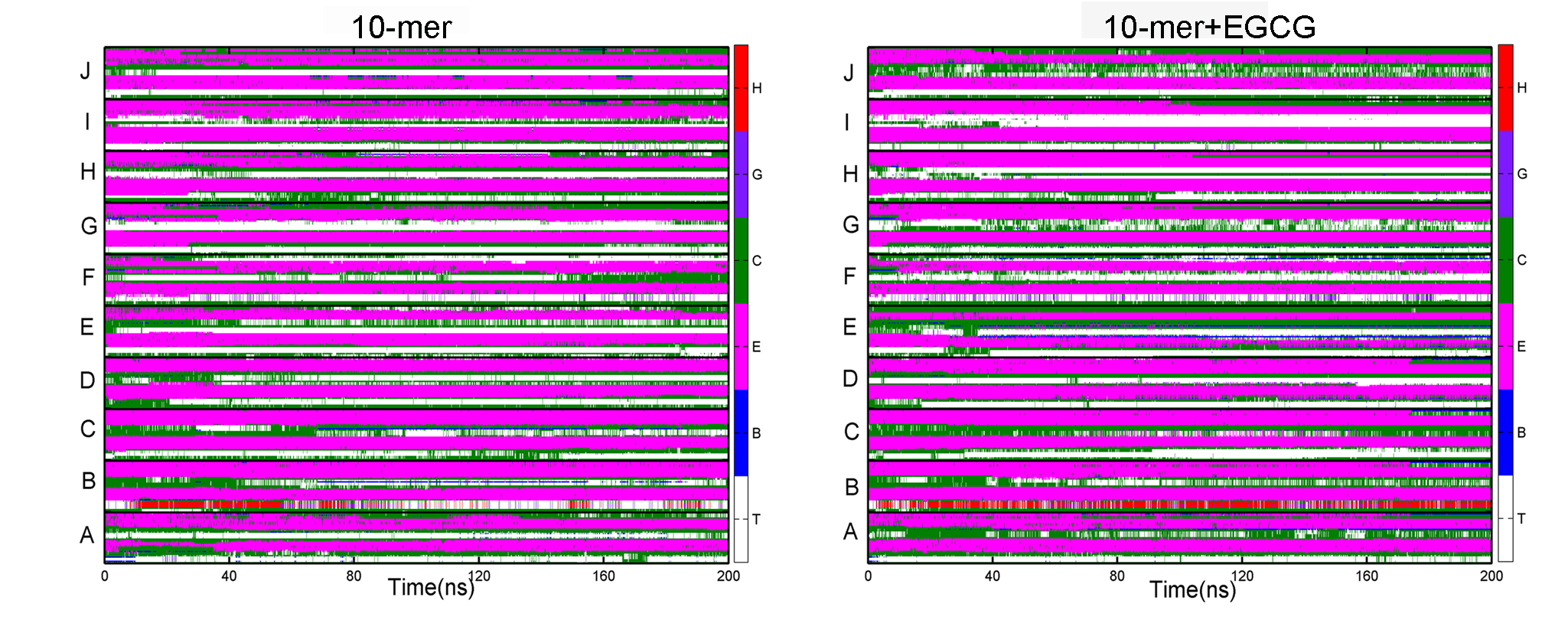

Supplement: Figure S1 — Time evolutions of the secondary structure of hIAPP1–37 10-mer calculated by STRIDE algorithm. Here, we label a turn by “T”, an isolated bridge by “B”, an extended conformation by “E”, a coil by “C”, a 310-helix by “G” and an α-helix by “H”. (TIF) [file pone.0094796.s001.tif]

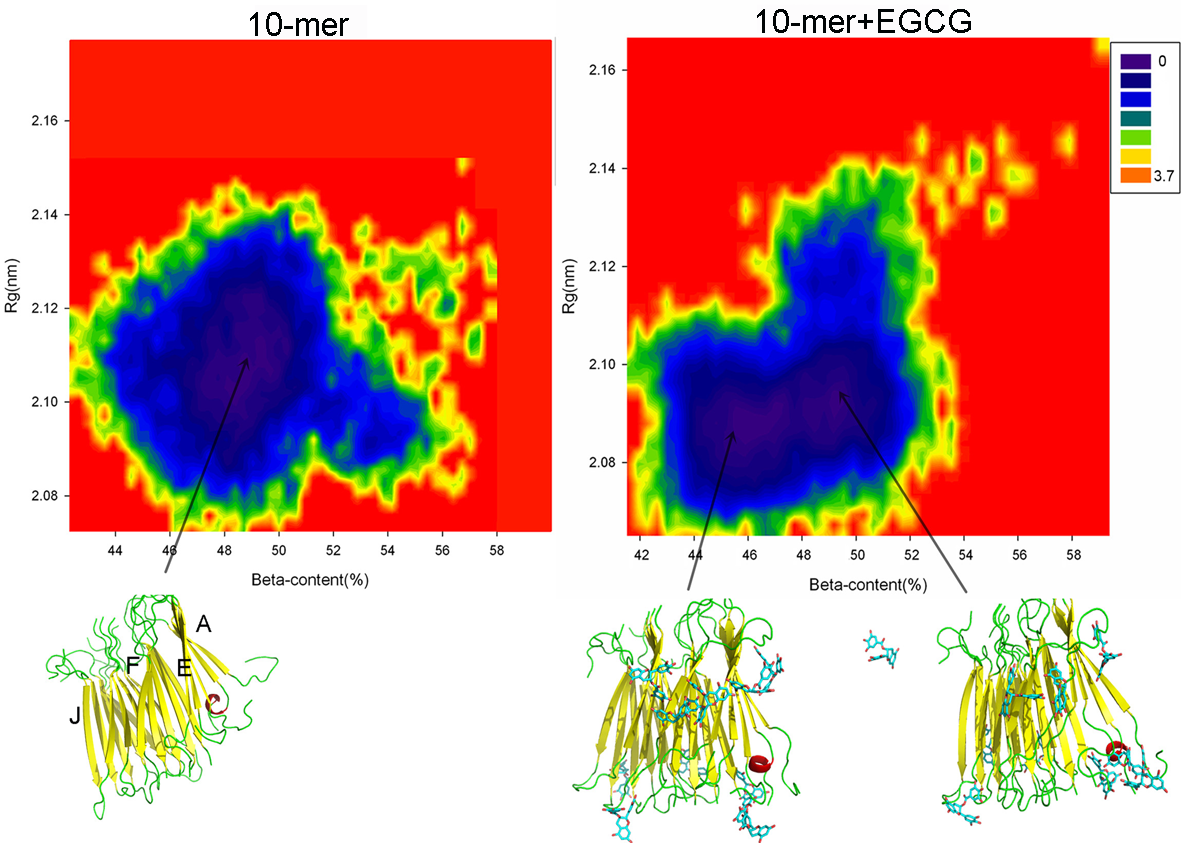

Supplement: Figure S2 — Free energy landscapes (in kcal mol−1) and lowest-free-energy structures at basins for hIAPP1–37 10-mer in the presence and absence of EGCG. (TIF) [file pone.0094796.s002.tif]

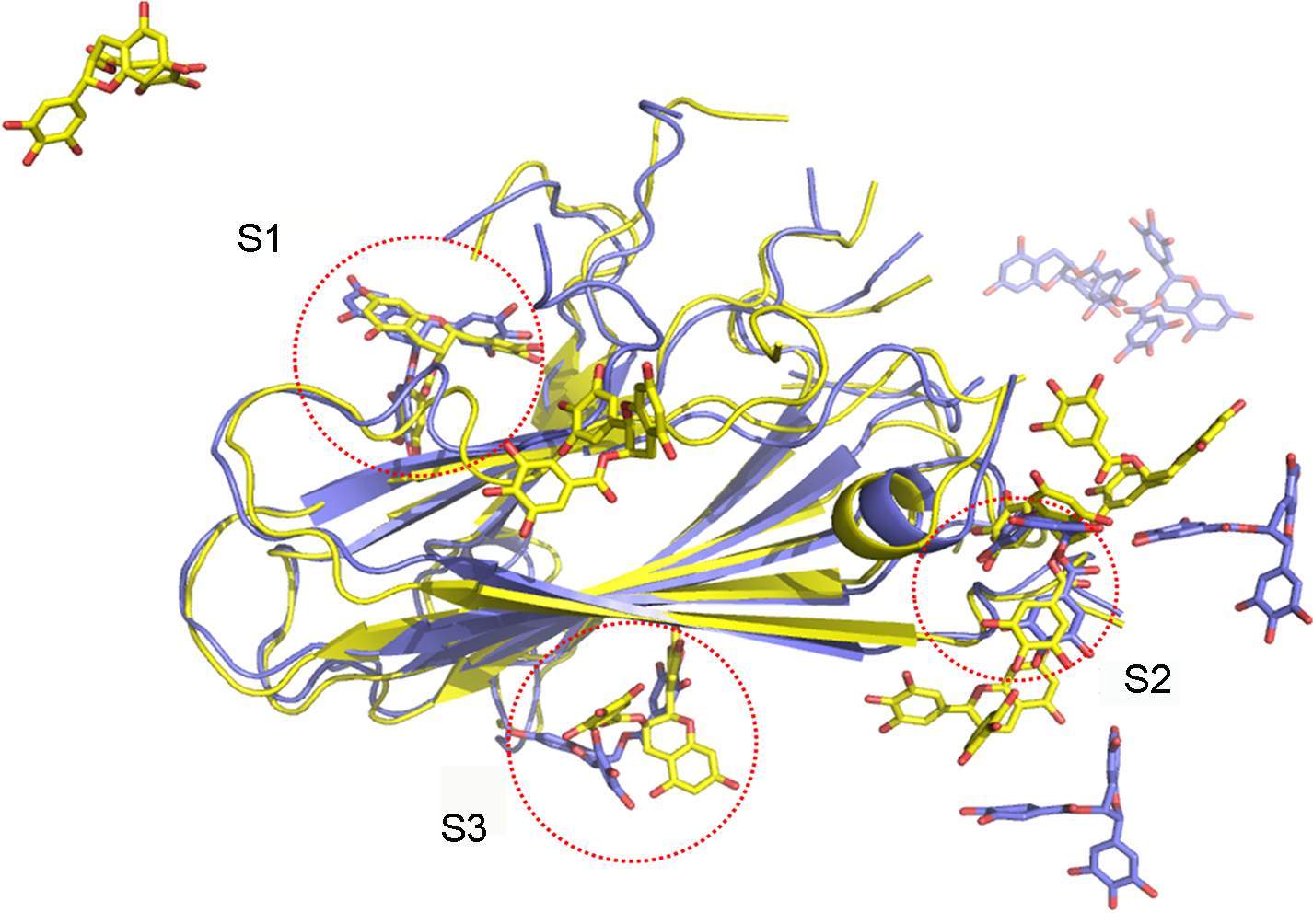

Supplement: Figure S3 — The aligned representative structures of the first two clusters. Violet and yellow cartoon corresponds to the representative structure of the first and second cluster, respectively. Three domains circled by dashed line are indicative of S1, S2 and S3 site. (TIF) [file pone.0094796.s003.tif]

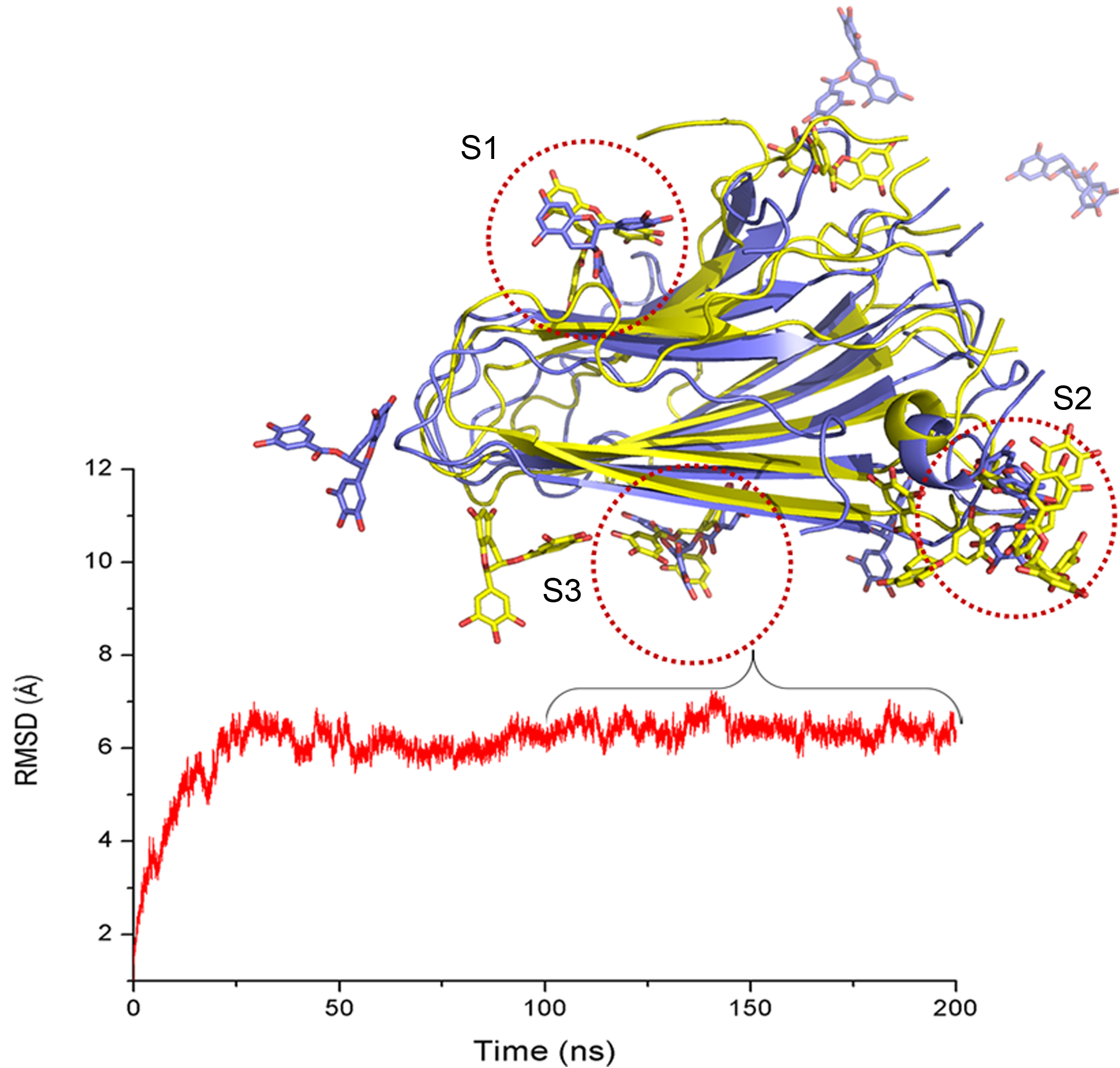

Supplement: Figure S4 — Time evolutions of RMSD values of hIAPP1–37 5-mer with EGCG for the parallel 200 ns trajectory together with the aligned representative structures of the first two clusters from cluster analysis (last 100 ns). (TIF) [file pone.0094796.s004.tif]
