# Supplementary material for: Exploring the Influence of EGCG on the β-Sheet-Rich Oligomers of Human Islet Amyloid Polypeptide (hIAPP1–37) and Identifying Its Possible Binding Sites from Molecular Dynamics Simulation
Source: PLoS One. 2014 Apr 16;9(4):e94796. doi: 10.1371/journal.pone.0094796 (PMC3989243; doi:10.1371/journal.pone.0094796)
Supplement: Table S1 — Names and types of atoms, charges and masses of EGCG used in the simulations. (DOC) [file pone.0094796.s005.doc]

**Table S1.** Names and types of atoms, charges and masses of EGCG used in the simulations.

| No. | Name | Type | Charge | Mass |
| --- | --- | --- | --- | --- |
| 1 | CAM | C2R61 | -0.140 | 12.011 |
| 2 | HAM | HGR61 | 0.140 | 1.008 |
| 3 | CAW | C2R61 | 0.203 | 12.011 |
| 4 | OAE | OG311 | -0.611 | 15.999 |
| 5 | HAE | HGP1 | 0.408 | 1.008 |
| 6 | CBC | C2R61 | 0.203 | 12.011 |
| 7 | OAI | OG311 | -0.611 | 15.999 |
| 8 | HAI | HGP1 | 0.408 | 1.008 |
| 9 | CAX | C2R61 | 0.203 | 12.011 |
| 10 | OAF | OG311 | -0.611 | 15.999 |
| 11 | HAF | HGP1 | 0.408 | 1.008 |
| 12 | CAN | C2R61 | -0.140 | 12.011 |
| 13 | HAN | HGR61 | 0.140 | 1.008 |
| 14 | CBA | C2R61 | 0.000 | 12.011 |
| 15 | CBG | CG311 | 0.200 | 12.011 |
| 16 | HBG | HGA1 | 0.090 | 1.008 |
| 17 | OAR | O3R60 | -0.580 | 15.999 |
| 18 | CBD | C2R61 | 0.290 | 12.011 |
| 19 | CAO | C2R61 | -0.140 | 12.011 |
| 20 | HAO | HGR61 | 0.140 | 1.008 |
| 21 | CAT | C2R61 | 0.203 | 12.011 |
| 22 | OAB | OG311 | -0.611 | 15.999 |
| 23 | HAB | HGP1 | 0.408 | 1.008 |
| 24 | CAJ | C2R61 | -0.140 | 12.011 |
| 25 | HAJ | HGR61 | 0.140 | 1.008 |
| 26 | CAY | C2R61 | 0.203 | 12.011 |
| 27 | OAG | OG311 | -0.611 | 15.999 |
| 28 | HAG | HGP1 | 0.408 | 1.008 |
| 29 | CBE | C2R61 | 0.000 | 12.011 |
| 30 | CAP | CG321 | -0.180 | 12.011 |
| 31 | HAP | HGA2 | 0.090 | 1.008 |
| 32 | HAQ | HGA2 | 0.090 | 1.008 |
| 33 | CBF | CG311 | 0.200 | 12.011 |
| 34 | HBF | HGA1 | 0.090 | 1.008 |
| 35 | OAQ | OG302 | -0.370 | 15.999 |
| 36 | CAS | CG2O2 | 0.630 | 12.011 |
| 37 | OAA | OG2D1 | -0.550 | 15.999 |
| 38 | CAZ | C2R61 | 0.000 | 12.011 |
| 39 | CAK | C2R61 | -0.140 | 12.011 |
| 40 | HAK | HGR61 | 0.140 | 1.008 |
| 41 | CAU | C2R61 | 0.203 | 12.011 |
| 42 | OAC | OG311 | -0.611 | 15.999 |
| 43 | HAC | HGP1 | 0.408 | 1.008 |
| 44 | CBB | C2R61 | 0.203 | 12.011 |
| 45 | OAH | OG311 | -0.611 | 15.999 |
| 46 | HAH | HGP1 | 0.408 | 1.008 |
| 47 | CAV | C2R61 | 0.203 | 12.011 |
| 48 | OAD | OG311 | -0.611 | 15.999 |
| 49 | HAD | HGP1 | 0.408 | 1.008 |
| 50 | CAL | C2R61 | -0.140 | 12.011 |
| 51 | HAL | HGR61 | 0.140 | 1.008 |
